# Dopaminergic neurodegeneration in *C. elegans* cultivated with *Porphorymonas gingivalis*

**DOI:** 10.17912/micropub.biology.001423

**Published:** 2025-01-06

**Authors:** Edward F. Griffin, Madeline G. Owens

**Affiliations:** 1 Natural Sciences, Converse University, Spartanburg, South Carolina, United States

## Abstract

Disruption of the human microbiome has emerged as a major contributing factor in the etiology of neurodegenerative disease. Previous work suggests a positive correlation between periodontal inflammation and Parkinson's disease. Here, we show that feeding *C. elegans* animals *Porphorymonas gingivalis* causes neurodegeneration that is not additive with neurodegeneration induced by the Parkinson's-associated protein, α-synuclein. In contrast, α-synuclein-expressing animals fed *P. gingivalis* show additional disruption in basal slowing, suggesting that *P. gingivalis* induces neurodegeneration while altering neuronal function of extant neurons. Though the mechanism is unclear, these results suggest a relationship between *P. gingivalis* and neurodegeneration that warrants further investigation.

**Figure 1. Cultivation of C. elegans with P. gingivalis results in neurodegeneration and impairs basal slowing response:**
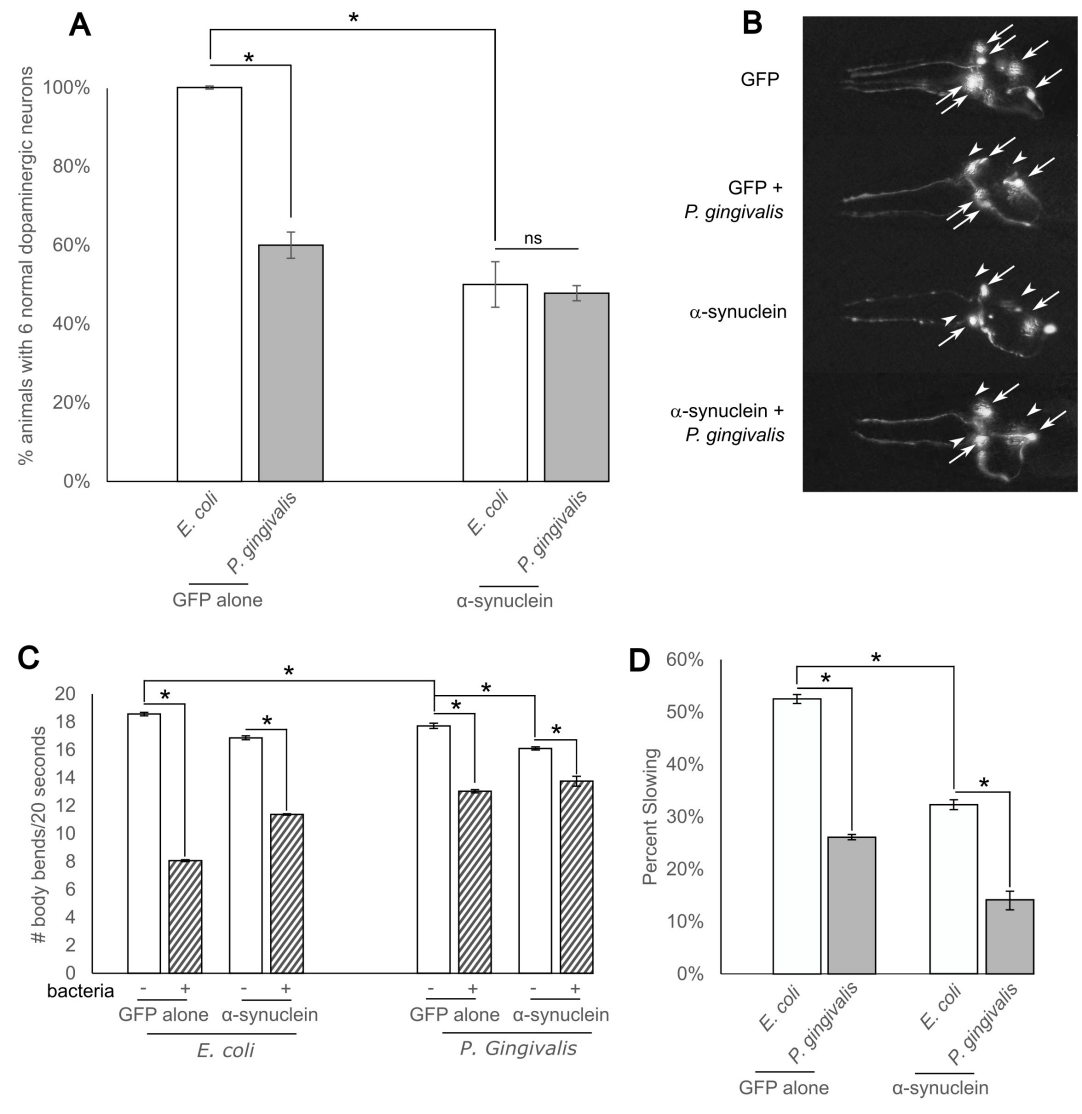
Cultivation of *C. elegans* with *P. gingivalis* induces neurodegeneration and reduces neuronal function[BM1] . (A) Animals were cultivated with either standard *E. coli* or *P. gingivalis* and analyzed for neurodegeneration 7 days *post* -hatching (n=30 animals analyzed in 3 replicates) (B) Representative images of neurodegeneration. Arrows indicate neurons while arrowheads indicate degenerating neurons or regions where neurons are missing. (C) Animals were cultivated with either standard *E. coli* or *P. gingivalis* and analyzed for basal slowing (n=30 animals analyzed in 3 replicates). (D) Percent slowing was calculated by dividing the difference in slowing between fed and unfed animals by the number of body bends/20s when fed. Data represented as mean and error bars represent standard deviation. *=p<0.001 according to One-Way ANOVA with a Tukey's *post hoc* test.

## Description

Parkinson's disease (PD) is the one of the most common neurodegenerative diseases, characterized by progressive neurodegeneration of the *substantia nigra pars compacta* (SNPC) and the formation of intracellular inclusions that contain insoluble α-synuclein (Kim et al., 2019; Prasad et al., 2023) . While the etiology of PD is not fully understood, it is clear that a multitude of genetic and environmental variables contribute to risk and onset. For example, multiplication of the α-synuclein locus results in autosomal-dominant Parkinson's disease (Polymeropoulos et al *.* , 1997; Singleton et al *.* , 2003) and single nucleotide polymorphisms (SNPs) in distinct loci may increase risk (Harrington et al., 2011; Griffin et al., 2018). Environmental factors, such as secondary metabolites of environmental microbes, have been shown to induce unfolded protein stress responses and mitochondrial damage (Bornhorst et al., 2014; Martinez et al., 2015, 2017b) that may also lead to disease (Kim et al., 2018) .

*Porphorymonas gingivalis* has been identified in postmortem analysis of both Parkinson's and Alzheimer's disease patients (Jungbauer et al., 2022) , suggesting a relationship between periodontal disease and neurodegenerative disease. Oral administration of *P. gingivalis* increases gut permeability, immune responses (Feng et al., 2020) , and dysbiosis of gut microbiota (Arimatsu et al., 2014; Nakajima et al., 2015) . Indeed, gut dysbiosis has been associated with Parkinson's disease (Campos-Acuña et al., 2019; Kim et al., 2019). These highlight the relationship between neurodegeneration and microbial invasion, which may be occurring from major arteries (Mougeot et al., 2017) .

While it is becoming increasingly evident that inflammation contributes to neurodegeneration (Gerhard et al., 2006; Brochard et al., 2009; Williams et al., 2021) , the effect of oral microbiota on neurodegeneration remains unclear. To examine whether *P. gingivalis* has a direct effect on neurodegeneration, we utilized a *C. elegans* model of α-synuclein toxicity. In this model, expression of α-synuclein in the dopaminergic neurons leads to progressive neurodegeneration of the 6 dopaminergic neurons of the head, which are observed by constitutive GFP expression (Martinez et al., 2017a). Because the *C. elegans* diet consists of bacteria, they can be fed *P. gingivalis* and examined for neurodegeneration. Previous work in this model has identified environmental microbial factors that affect neurodegeneration (Martinez et al., 2015, 2017b), demonstrating its utility in examining the cellular and molecular effects of environmental microbes on neurodegeneration. Additionally, because *C. elegans* have neither vasculature nor NF-κB, we can examine neurodegeneration caused by bacteria apart from stereotypical hallmarks of inflammation. We therefore hypothesized that feeding *P. gingivalis* to *C. elegans* animals would result in neurodegeneration and that neurodegeneration would be exacerbated by expression of α-synuclein.

In a previous study, animals were treated with *Streptomyces* spp.-conditioned media and examined for neurodegeneration (Caldwell et al., 2009) . After 6 days of exposure, about 25% of animals exposed to *S. venezuelae* exhibited neurodegeneration, while neurodegeneration was observed in only 6% and 7% of animals exposed to *S. griseus* and *S. coelicolor* media, respectively. To determine whether *P. gingivalis* alone induces neurodegeneration, we first examined the effect of *P. gingivalis* on animals expressing GFP alone. As expected, 100% of wild-type GFP-expressing animals cultivated on standard *E. coli* ( OP50 ) exhibited all 6 dopaminergic neurons, however, when cultivated on lawns of *P. gingivalis* , only about 60% of wild-type GFP-expressing animals exhibited all 6 dopaminergic neurons ( Figure 1A and B).

To examine whether the effect of *P. gingivalis* were synergistic with α-synuclein, we utilized a *C. elegans* strain co-expressing α-synuclein in the dopaminergic neurons. When cultivated on OP50 , only about 50% of animals co-expressing α-synuclein exhibit all 6 dopaminergic neurons ( Figure 1A and B). However, when animals co-expressing α-synuclein were cultivated on *P. gingivalis* , there was no statistically significant difference in neurodegeneration compared to α-synuclein co-expressing animals cultivated on OP50 , suggesting the neurodegenerative effects of *P. gingivalis* and α-synuclein are neither additive nor synergistic, perhaps operating through similar cellular pathways.

It is possible that secondary metabolites secreted by *P. gingivalis* have similar cellular targets as α-synuclein. For example, nematodes over-expressing α-synuclein exhibit dysfunctions in mitochondrial DRP-1 (Martinez et al., 2018) and infection of *P. gingivalis* in mice results in mitochondrial dysfunction through Drp-1 (Xu et al., 2021) . Similarly, secretion of a secondary metabolite by *Streptomyces venezuelae* increases *drp-1* expression and may increase cell death through DRP-1 (Kim et al., 2018) . Gingipains secreted by *P. gingivalis* have been observed in the bloodstream of some Parkinson's disease patients (Adams et al., 2019) . Some gingipains have been observed to bind to mitochondria (Boisvert and Duncan, 2010) , promoting mitochondrial dysfunction through Drp1 (Xu et al., 2021) . However, if it were the case that *P. gingivalis* were causing neurodegeneration in *C. elegans* through DRP-1 , we would expect to see an additive effect of neurodegeneration such as was observed in *S. venezuelae* (Martinez et al., 2015) . Indeed, the gingipain adhesion protein RgpA binds to host mitochondria to block apoptosis (Boisvert and Duncan, 2010) , whereas *S. venezuelae* appears to stimulate apoptosis. In contrast, gingipains from the *P. gingivalis* strain W83 disrupt cell adhesion and promote apoptosis by cleavage of host cadherins (Sheets et al., 2006) .

While we observed no additive effects of *P. gingivalis* and α-synuclein on neurodegeneration, it is possible that surviving neurons may nonetheless exhibit loss of function. This phenomenon has been demonstrated in other models, such as with loss of function despite neuroprotection in a glutamatergic model of β-amyloid and ApoE co-expression (Griffin et al., 2019) . To test this, we examined basal slowing, which is influenced by the dopaminergic system (Sawin et al., 2000) . When unfed, nematode locomotory rate is enhanced; upon encountering food, locomotion is depressed. Thus, perturbations in dopaminergic signaling affected basal slowing. When encountering *E. coli* , locomotory rate typically decreases. In contrast, when fed *Bacillus subtilis* , animals appeared to increase locomotory frequency, while there is no change when fed *Pseudomonas aeruginosa* (Rivard et al., 2010) . Animals cultivated on *P. gingivalis* had a statistically significant lower basal locomotory rate compared to animals cultivated on OP50 ( Figure 1C ). More specifically, when examining the percent slowing between animals off food and on food, α-synuclein co-expressing animals had a significantly smaller percent slowing in basal slowing when cultivated on *P. gingivalis* when compared to OP50-fed animals ( Figure 1D ), suggesting an additive effect on loss of dopaminergic function for surviving neurons. Thus, basal slowing was perturbed in all populations cultivated on *P. gingivalis* compared to animals cultivated on OP50 .

Some gingipains target the endolysosomal system by exploiting clathrin-dependent endocytosis (Boisvert and Duncan, 2008) . Disruptions in the endolysosomal system increase neurodegeneration in Parkinson's models (Harrington et al., 2011; Griffin et al., 2018) and disruptions in clathrin-dependent endocytosis increase neurodegeneration in Alzheimer's models of disease (Treusch et al., 2011; Griffin et al., 2018) . Additionally, perturbations in the endolysosomal system may also affect the recycling of dopamine receptors at the cell surface. Recycling of D1 receptors is enabled through a C-terminal recycling signal with homology to β _2_ adrenergic receptors and μ opioid receptors (Vargas and Von Zastrow, 2004) . Interference with the C-terminal region results in enhanced signaling (Cao et al., 1999; Tanowitz and von Zastrow, 2003) , demonstrating D1 receptor expression at the plasma membrane can be impaired by endocytosis. Alternatively, disruptions in endocytosis may affect recycling of the dopamine active transporter (DAT) at the cell surface. For example, a previous study found that a non-functional mutation in RAB39B, which localizes to the Golgi and early endosomes to coordinate recycling of DAT-1 in *C. elegans* , decreased dopaminergic signaling when probing cholinergic behavior modulated by dopaminergic signaling (Zeng et al., 2024) . Thus, gingipains may diminish dopaminergic signaling at the cell surface by stimulating endocytosis of receptors or DAT.

While the cause of Parkinson's disease is not clear, it is evident that multiple genetic and environmental factors contribute to its etiology. Between 2011 and 2020, the prevalence of periodontitis was estimated to be about 62% in adults and severe periodontitis is estimated to be 23.6% globally (Trindade et al., 2023) . These numbers are alarming, particularly considering some reports have identified a positive correlation between periodontal inflammation and Parkinson's disease (Yilmaz et al., 2023) . As such, the risk of *P. gingivalis* should not be overlooked, considering observations of microbial presence in the arteries and brain. Here, we show that exposure to *P. gingivalis* directly causes neurodegeneration in an *in vivo* model of neurodegeneration. Taken together, these results suggest that the relationship between *P. gingivalis* periodontal disease and Parkinson's disease warrants further investigation.

## Methods

**Culture Preparation and *C. elegans* Husbandry**

*E. coli* ( OP50 ) was prepared by inoculating LB broth and incubated overnight at 37°C. *P. gingivalis* strain W83 was acquired from ATCC and cultured in New Oral Spirochete Broth (NOS; ATCC medium 1494) in an anaerobic chamber with a gas pack and incubated with shaking at 37°C up to overnight.

GFP-expressing animals ( BY250 [ *vtIs7* ( *dat-1* p:: *GFP* )]) and α-synuclein co-expressing animals ( UA44 [ *baIn11* (P *_dat-1_* :: *α-syn* , P *_dat-1_* :: *GFP* )]) were a generous gift from Dr. Guy Caldwell. *C. elegans* hermaphrodite animals were maintained on *E. coli* lawns, according to standard procedures (Brenner, 1974) , and synchronized populations were analyzed for neurodegeneration at day 7 *post* -hatching, unless otherwise indicated. Briefly, animals for analysis were synchronized by a 3 h egg-lay on bacterial lawns containing either standard *E. coli* cultures (as a control), or *P. gingivalis* . Animals were examined for dopaminergic neurodegeneration at day 7 *post* -hatching.

**Neuron Analysis**

Animals were immobilized using 3mM levamisole on glass cover slips, inverted onto 2% agarose pads on microscope slides, and observed by fluorescent microscopy. An animal was scored as normal if all 6 dopaminergic neurons in the head were present and without malformations such as neurite blebbing, cell body rounding, cell loss, or dendrite or axon loss. Each analysis was replicated at least three times with 30 animals per condition (30 animals × 3 trials = 90). Images were acquired using an Olympus BX41 equipped with a FITC filter cube and MoticamX camera driven by Motic Images Plus 3.1.

**Basal Slowing Response Assay**

Basal slowing response was performed as previously described (Sawin et al., 2000) , with few modifications. The number of body bends in 20 seconds was recorded for well-fed animals and starved animals as they entered a bacterial lawn. Briefly, 30 well-fed animals were removed from plates, washed twice in S basal buffer, and transferred to the center of a locomotory assay plate. This plate has ring-shaped bacterial lawn and animals are transferred to the center of the clear zone within the ring. Five minutes after transfer, the number of body bends in 20 seconds was recorded for each of the animals on the plate as they entered the bacterial lawn. To test starved animals, 30 animals were washed twice in S basal buffer, transferred to NGM plates with no bacteria, and incubated for 30 minutes. After food deprivation, animals were washed off the plates and transferred to the center of an assay plate seeded with OP50 bacteria to measure locomotory rate similar to previously described (Sawin et al., 2000) . Percent slowing was calculated by dividing the difference in slowing between fed and unfed animals by the number of body bends/20s when fed.

**Table:** 

Table 1. Strains used in this study.		
Strain	Genotype	Source
BY250	*vtIs7* [P *_dat-1_* :: *GFP* ]	Martinez, 2017
UA44	*baIn11* [P *_dat-1_* :: *α-syn* , P *_dat-1_* :: *GFP* ]	Martinez, 2017
